# C/EBPβ–TFAM-Mediated NLRP3 Inflammasome Activation Contributes to Arsenic-Induced Rat Kidney Injury

**DOI:** 10.3390/toxics11080668

**Published:** 2023-08-02

**Authors:** Ziqin Wu, Wenjuan Wang, Kai Zhu, Daopeng Luo, Aihua Zhang

**Affiliations:** 1 The Key Laboratory of Environmental Pollution Monitoring and Disease Control, Ministry of Education, Department of Toxicology, School of Public Health, Guizhou Medical University, Guiyang 550025, China; wuziqin152@163.com (Z.W.); reality0337@126.com (W.W.); 18275101004@163.com (K.Z.); luodaopeng@gmc.edu.cn (D.L.); 2 Collaborative Innovation Center for Prevention and Control of Endemic and Ethnic Regional Diseases Co-Constructed by the Province and Ministry, Guizhou Medical University, Guiyang 550025, China

**Keywords:** arsenic, rats, kidney, inflammation, NLRP3

## Abstract

Compelling evidence has demonstrated that arsenic (As) exposure is associated with kidney injuries. Given that inflammatory responses and immune imbalances are the root causes of several kidney diseases, this study investigated the potential mechanisms underlying NLRP3 inflammasome activation in As-induced kidney injury. A rat model of sub-chronic As exposure was established via oral administration of NaAsO_2_. The results revealed that urinary β-2-microglobulin (β2-MG), N-acetyl-β-D-glucosidase (NAG) and albumin (ALB) were increased in the As-exposed group, reflecting kidney impairment. Moreover, significant glomerular vacuole-like changes, tubular dilatation and inflammatory cell infiltration were observed. Meanwhile, the expression levels of neutrophil gelatinase-associated lipocalin (NGAL), IL-1β and IL-18 were enhanced in the kidney tissues of As-treated rats. Further, increased expression of NLRP3, ASC and caspase-1, which are NLRP3 inflammasome-associated proteins, were observed in the kidney tissues of rats in the As-treated groups. The expression levels of the NLRP3 upstream regulators C/EBPβ and TFAM were also elevated. These findings suggest that sub-chronic As exposure triggers inflammatory responses in rat kidney tissue and impairs kidney function. The underlying mechanisms may be related to the C/EBPβ–TFAM pathway and activation of the NLRP3 inflammasome pathway.

## 1. Introduction

Arsenic (As) is a notorious environmental contaminant. As concentrations in the groundwater of more than 35 countries around the world have surpassed the World Health Organization (WHO) standard of 10 μg/mL [1]. Prolonged exposure to As can result in a number of adverse health outcomes, such as multi-organ and multi-system damage, including damage to the liver, skin, lungs and kidneys [2]. The kidneys are an important organ for biotransformation and excretion and are a target organ for As toxicity [3]. Methylated compounds of As are less toxic and excreted more rapidly in urine than organic As [4]. As and its compounds are excreted from the body primarily through urine via the kidneys, making kidney damage unavoidable. Kidney injury can affect the excretion of As, leading to increased accumulation of As in the kidney system. Subsequently, proximal tubular and glomerular dysfunction can occur, which increases the risk of kidney injury [5]. Epidemiological evidence from Southeast Asia, Taiwan and several Western countries has confirmed that excessive As intake is a prominent environmental risk factor for kidney disease [3,6,7]. Kidney injury caused by As exposure leads to kidney cancer, proteinuria, hypercalciuria and a series of changes at the cellular and subcellular levels [8]. Although numerous theories have been proposed as to the mechanisms of As pathogenesis, including the oxidative stress theory, inflammatory response theory and apoptosis theory, the molecular mechanisms of As-induced kidney injury remain unclear.

Inflammation underlies several kidney diseases and causes glomerulosclerosis, tubular atrophy and fibrosis. This results in end-stage kidney disease, depending on the extent and duration of injury [9]. Inflammasomes are large multimeric protein complexes primarily found in innate immune cells. They respond to exogenous pathogens and endogenous danger signals, triggering an inflammatory response. The nucleotide-binding oligomerization domain (NOD)-like receptor protein 3 (NLRP3) inflammasome is the most characterized inflammasome [10]. It is a multiprotein complex consisting of NLRP3, apoptosis-associated speckle-like protein (ASC) and cysteine aspartate-specific protease (caspase-1). ASC is a linker protein containing a structural pyrin domain (PYD) that communicates with NLRP3 and a caspase activation and recruitment domain (CARD) that communicates with caspase-1 [11,12]. The NLRP3 inflammasome can activated by a series of damage-associated molecular patterns (DAMPs) and pathogen-associated molecular patterns (PAMPs), and it plays a critical role in the innate immune system [11]. The caspase-1 precursor contains CARD, p20 large subunit and p10 small subunit which, upon activation, cleave to p20 and p10, promoting the maturation and release of inflammatory parameters such as interleukin 1β (IL-1β) and interleukin 18 (IL-18). This induces the inflammatory response and apoptosis [13]. Previous studies found that As can promote activation of the NLRP3 inflammasome and generate an inflammatory response [14]. However, the molecular mechanism underlying the role of the NLRP3 inflammasome in As-induced kidney injury is yet to be clearly defined.

Mitochondrial transcription factor A (TFAM) is an important factor which is involved in the activation of mitochondrial DNA (mtDNA) transcription and the regulation of the copy number of mitochondrial DNA. TFAM is encoded by a nuclear gene and is transferred into mitochondria to play a regulatory role. It has been demonstrated that aberrant TFAM expression can lead to mtDNA deletion and severe respiratory chain deletion. Moreover, mitochondrial dysfunction in kidney tubular epithelial cells is one of the major triggers of multiple cell death pathways and inflammatory responses [15]. Our previous population-based epidemiologic studies revealed that As exposure significantly increases peripheral blood mtDNAcn, suggesting that TFAM may be a key target for As-induced adverse health outcomes and systemic inflammation [16,17]. Further, TFAM is a high-mobility-group (HMG) protein that is usually located in mitochondrial membranes, tissues or cells [18]. It shares a common site of proinflammatory activity with HMGB1, as they are structurally and functionally homologous [19]. HMGB1 can be released from damaged cells and promotes thermal degeneration. Alternatively, TFAM can be recognized by Toll-like receptors (TLRs) and receptors of advanced glycation endproducts (RAGE) because of its secretion from inflamed necrotic cells, which subsequently causes local and systemic inflammation [20]. The expression level of TFAM, as a functional protein, can also be regulated through various transcription factors via binding to its gene promoter [21]. CCAAT/enhancer-binding protein beta (C/EBPβ) is a transcription factor with highly conserved DNA binding and dimerization domains at the C-terminus. Its function is regulated by numerous pathways, including protease degradation, phosphorylation and protein interactions [22]. However, the effect of C/EBPβ on TFAM in As-induced kidney injury in rats is yet to be understood.

This study evaluated the potential mechanisms underlying NLRP3 inflammasome activation in As-induced kidney immune dysfunction by studying a sub-chronic rat model of As exposure. Immune inflammation in the kidneys was investigated by analyzing alterations in pathological and biochemical markers indicative of As-induced kidney injury and inflammation. The underlying mechanisms were evaluated with a focus on C/EPBβ, TFAM and NLRP3. The overarching goal of this research was to offer novel insights into the mechanisms of As-induced kidney injury.

## 2. Materials and Methods

### 2.1. Animal Models of Sub-Chronic Arsenic Treatment

**Figure 1:**
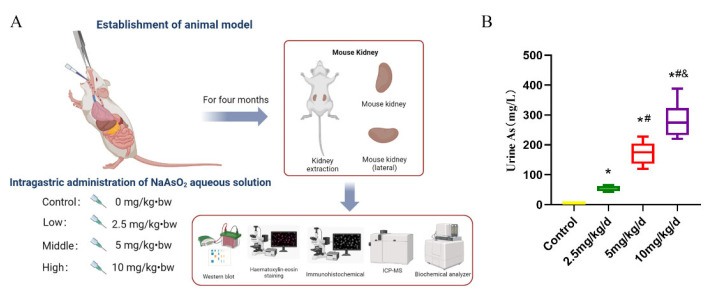
Establishment of As exposure rat model. (**A**) Schematic workflow of the As treatment in rat. (**B**) The urinary As concentration of rats in different groups. * *p* < 0.05 compared with the control group, # *p* < 0.05 compared with the 2.5 mg/kg/d NaAsO_2_ group, & *p* < 0.05 compared with the 5 mg/kg/d NaAsO_2_ group. The data are separated by median and quartile (*n* = 6).

Wistar rats (weanlings with a body weight of 80–100 g) were obtained from Guizhou Medical University (License number SCXK-LN2015-0003). Under standard experimental conditions, 24 weaned Wistar rats were randomly divided into four groups with equal numbers of males and females (a control group and three separate groups receiving doses of 2.5 mg/kg/d, 5 mg/kg/d and 10 mg/kg/d NaAsO_2_, respectively). The control group received deionized water through intragastric administration and, in line with our previous study [23], the As-treated groups received NaAsO_2_ intragastric administration for four months (Figure 1A).

The LD_50_ for acute oral toxicity of arsenic trioxide is 43.0 mg/kg/d. The chosen treatment concentrations were optimized based on the dosage design principles for sub-chronic toxicity. That is, 1/20, 1/10 and 1/5 of the LD_50_ were selected as the NaAsO_2_ concentrations for the low-, medium- and high-As-exposure groups (2.5, 5, and 10 mg/kg/d), respectively. Following dosage selection, a number of preliminary experiments were performed and the results indicated that exposure to As at these doses for four months did not kill the animals but did cause severe organ damage [23].

Throughout this research, the rats were housed in a conditioned environment (24 ± 1 °C, 60–70% relative humidity) under a cyclical light/dark cycle. All experimental steps involving animals were approved by the Animal Experimentation Ethics Committee of Guizhou Medical University (No. 2000868).

### 2.2. Sample Collection

Metabolic cages were used to collect 24-h urine from rats. The rats were given sufficient water before the experiment and prohibited from drinking during the experiment. Due to the small amount of urine excreted by rats, the loss and evaporation that occur during manipulation, and the inconsistency in bladder emptying among rats, large measurement error can occur. Therefore, 24-h urine was collected in this study. Hydrochloric acid was added to the samples to acidify the urine before the determination of urinary As. Urine alkalized with sodium hydroxide was used for Beta-2 microglobulin (β2-MG) determination, and untreated raw urine was used for urinary albumin (ALB) and N-acetyl-beta-D-glucosaminidase (NAG) determination. The rats were anesthetized with 0.9% pentobarbital sodium (Sigma, St. Louis, MA, USA) (150 mg/kg), and their kidneys were isolated immediately. The left kidney was fixed in 4% formalin for 24 h and then embedded in paraffin for hematoxylin–eosin (HE) staining and immunohistochemical (IHC) studies. The right kidney was frozen in liquid nitrogen and preserved for Western blot (Wb) analysis.

### 2.3. Determination of Urine As

Urine samples frozen and stored at −80 °C in a refrigerator were thawed and mixed at room temperature. The samples were acidified at a urine:ultrapure nitric acid ratio of 20:1. The acidified urine samples were then diluted 15 times with 1% nitric acid solution and mixed thoroughly. The supernatant was then obtained for the detection of urinary As concentration. The urinary As concentration was detected by an inductively coupled plasma mass spectrometer (ICP-MS, NexION 2000, PerkinElmer, Waltham, MA, USA). Before detection, the internal standard solution, standard curve solution and blank control solution (1% nitric acid) were configured. The detection mode of the instrument was set to collision mode. The original urinary As concentration was calculated based on the dilution of the standard curve and the urine sample before processing.

### 2.4. Determination of Kidney Function

An β2-MG kit, purchased from Nanjing Jiancheng (E015-1-1, Nanjing, China), was used for the detection of kidney function, and the detection limit was 0.2–18 mg/L. The NAG kit was purchased from Nanjing Jiancheng (A031-1-1, Nanjing, China), and the detection limit was 0.1–80 U/L. The ALB kit was purchased from Nanjing Jiancheng (A028-2-1, Nanjing, China), and the detection limit was 5–1000 mg/mL.

### 2.5. HE Staining

Kidney tissues were immobilized in formalin and then embedded in paraffin. Next, the sections (5-μm) were deparaffinized in xylene and hydrated with gradient concentrations of alcohol. They were then dyed with HE solution and rinsed with running water. Next, the sections were dipped in gradient ethanol for dehydration, cleared in xylene, dried naturally, sealed with neutral gum and covered with coverslips. The final products were observed under a microscope.

### 2.6. Western Blot Analysis

Cryopreserved rat kidney tissues were placed into RIPA lysis buffer (Beyotime, Shanghai, China) and ground with zirconia beads. The concentration of protein was determined with a BCA protein detection kit (Beyotime, Shanghai, China) after taking the supernatant. The protein was diluted to 2 μg/μL and denatured at 100 °C for 10 min. The extracted protein was separated using 10% or 12% polyacrylamide gel electrophoresis (PAGE) and transferred to a polyvinylidene fluoride membrane. The membrane was incubated with primary antibody working solution and all antibodies were purchased from Absin, China; the dilution ratio was 1:1000. Next, the membrane was incubated with the diluted secondary antibody working solution (anti-immunoglobin horseradish peroxidase-linked antibody) at a ratio of 1:8000 for 60 min. After cleaning, an enhanced chemiluminescence (ECL, Smart-Lifesciences, Changzhou, China) kit was used to detect the immune complexes. The bands of target proteins were analyzed using Image J 1.8.0.112 software, and blots were quantified by densitometry. The results were normalized by dividing the relative protein expression of the As-treated group by the control group.

### 2.7. IHC Analysis

The sections were deparaffinized in xylene and hydrated in gradient concentrations of alcohol. After the sections were immersed in citrate buffer for antigen repair, 3% H_2_O_2_ was added to quench endogenous peroxidase. Goat serum was added to block non-specific antigen binding. After discarding the serum, the sections were incubated with primary antibody working solution at 4 °C overnight; the dilution ratio was 1:200. After PBS cleaning, biotinylated secondary antibody (goat anti-rabbit, 2.0 μg/mL) was added to detect the bound antibodies. Diaminobenzidine (DAB, Amresco^®^, Framingham, MA, USA) was used to enhance the reaction for 30 s, and the sections were counterstained with hematoxylin; a differentiation solution was used for differentiation. The sections were then dehydrated in gradient ethanol, cleared in xylene, dried naturally, sealed with neutral gum and covered with coverslips. The immunoreactive positive expression in dense areas was observed using a light microscope and digital camera starting at a low magnification for each slide and then at 400× magnification. The immunoreactivity of the target antigen was quantified by Image-Pro-Plus 6.0.0.260 software.

### 2.8. Statistical Analysis

Data analysis was performed by SPSS 25.0 statistical software. Before analyzing the data, it was first verified that the data from all groups were normally distributed. If the data were normally distributed, a parametric test was performed; otherwise, a non-parametric test was performed. Continuous variables and normally distributed data were analyzed by one-way analysis of variance. For further pairwise comparison, the Bonferroni test was used for homogeneous variances, and the Tamhane’s T2 test was used for uneven variances. The non-normal data were analyzed by a Kruskal–Wallis H test. Spearman correlations were performed for correlation analysis. Statistical significance was set at α = 0.05.

## 3. Results

### 3.1. As Treatment Induced Histopathological Structural Changes and Kidney Dysfunction

**Figure 2:**
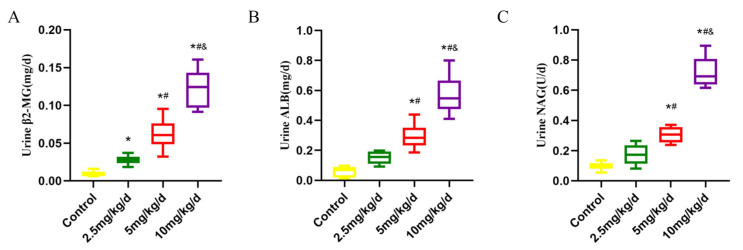
The daily excretion of β2-MG, ALB and NAG of the rats in different groups. (**A**) β2-MG (β-2-microglobulin), (**B**) ALB (albumin), (**C**) NAG (N-acetyl-β-D-glucosidase). * *p* < 0.05 compared with the control group, # *p* < 0.05 compared with the 2.5 mg/kg/d NaAsO_2_ group, & *p* < 0.05 compared with the 5 mg/kg/d NaAsO_2_ group. The data are separated by median and quartile (*n* = 6).

The results revealed that As treatment significantly increased the concentration of As in rat urine, with a dose–response relationship (*p* < 0.05, Figure 1B). Furthermore, the daily excretion of β2-MG, ALB and NAG were significantly increased in the urine of rats after As treatment, with a dose–response relationship (*p* < 0.05, Figure 2A–C).

**Figure 3:**
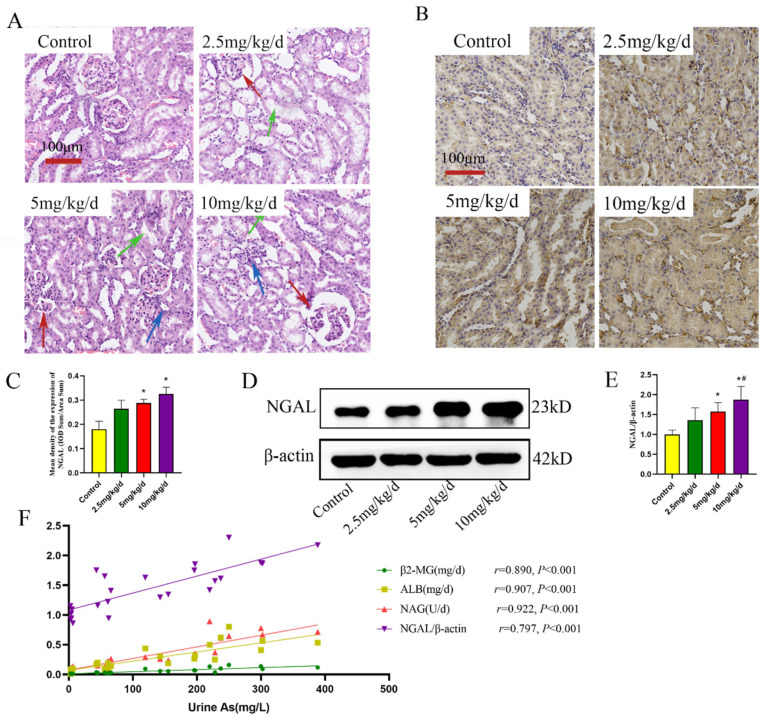
As exposure induced kidney impairment in rats. (**A**) Morphological changes in the kidney tissue (red arrow: glomerular vacuolation; green arrow: interstitial lymphocyte infiltration; blue arrow: interstitial lymphocyte infiltration). (**B**) Immunohistochemical detection of NGAL (neutrophil gelatinase-associated lipocalin). (**C**) Quantitative analysis of NGAL immunohistochemical detection. (**D**) Western blot detection of NGAL. (**E**) Quantitative analysis of NGAL Western blot detection. (**F**) Spearman correlation analysis of kidney function parameters and urinary As concentration. * *p* < 0.05 compared with the control group, # *p* < 0.05 compared with the 2.5 mg/kg/d NaAsO_2_ group. Data are reported as means ± SD (*n* = 6).

The effect of As on kidney histomorphology was assessed using HE staining. Under light microscopy, the kidney tissues of the control rats were intact with uniform staining. However, the rats treated with As had glomerular vacuolization, tubular dilatation or swelling and interstitial lymphocyte infiltration (Figure 3A). The expression levels of NGAL in the kidney tissues were detected by Wb and IHC. The immunohistochemical results revealed that NGAL expression was rare in the kidney tubules of the control rats. In contrast, NGAL expression was elevated in the kidney tissues of the As-treated rats, primarily in the cytoplasm of the kidney tubule epithelial cells (*p* < 0.05, Figure 3B,C). Similarly, the Western blot results revealed that NGAL expression was also considerably increased (*p* < 0.05, Figure 3D,E). Spearman correlation analysis revealed that the urine concentrations of β2-MG, ALB and NAG increased with increases in urinary As concentration, and the NGAL expression level in the kidney tissue was positively associated with the urinary As concentration (*p* < 0.05, Figure 3F).

### 3.2. As Induced Inflammation in the Rat Kidney

**Figure 4:**
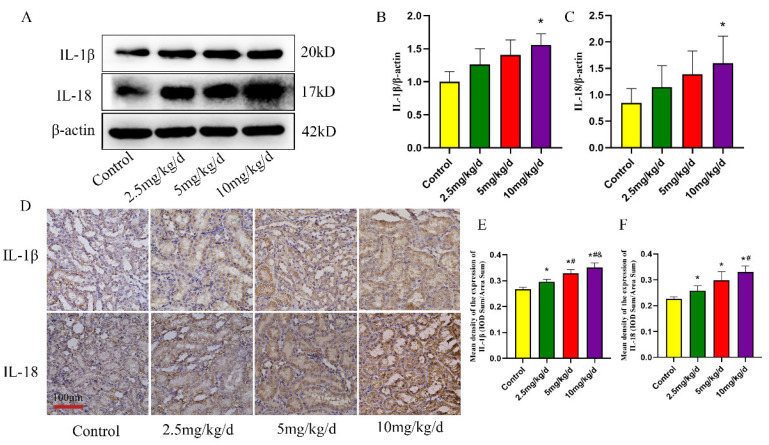
As-induced inflammation in the rat kidney. (**A**) Western blot detection of IL-1β and IL-18 expression. (**B**,**C**) Quantitative analysis of IL-1β and IL-18 protein expression, respectively. (**D**) Immunohistochemical detection of IL-1β and IL-18. (**E**,**F**) Quantitative analysis of IL-1β and IL-18 immunohistochemical detection, respectively. * *p* < 0.05 compared with the control group, # *p* < 0.05 compared with the 2.5 mg/kg/d NaAsO_2_ group, & *p* < 0.05 compared with the 5 mg/kg/d NaAsO_2_ group. Data are reported as means ± SD (*n* = 6).

The expression of inflammatory parameters was examined to verify the inflammatory damage in rat kidney tissue due to As treatment. The findings revealed significant increases in the expression of IL-1β and IL-18 proteins with increases in the As concentration (*p* < 0.05, Figure 4A–C). The localized expression of inflammatory parameters was further assessed by IHC. The results revealed that IL-1β and IL-18 were primarily expressed in kidney tubular epithelial cells and were significantly increased by As treatment (*p* < 0.05, Figure 4D–F).

### 3.3. As-Induced NLRP3 Inflammasome Activation in the Rat Kidney

**Figure 5:**
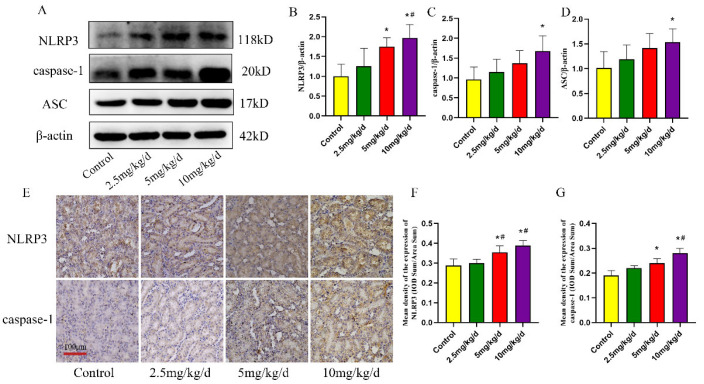
Expression of the NLRP3 inflammasome in the rat kidney. (**A**) Western blot detection of NLRP3, caspase-1 and ASC expression. (**B**–**D**) Quantitative analysis of NLRP3, caspase-1 and ASC protein expression, respectively. (**E**) Immunohistochemical detection of NLRP3 and caspase-1. (**F**,**G**) Quantitative analysis of NLRP3 and caspase-1 immunohistochemical detection, respectively. * *p* < 0.05 compared with the control group, # *p* < 0.05 compared with the 2.5 mg/kg/d NaAsO_2_ group. Data are reported as means ± SD (*n* = 6).

The NLRP3 inflammasome plays a crucial role in innate immunity. In order to investigate its role in As-induced kidney injury and the underlying mechanism, the expression of NLRP3 inflammasome-related molecules in kidney tissues were detected. The results demonstrated that the expression levels of NLRP3, caspase-1 and ASC in the kidney tissues of the As-exposed groups were upregulated (*p* < 0.05, Figure 5A–D). As expected, the IHC results revealed that the expression levels of NLRP3 and caspase-1 in the kidney tissues of rats exposed to As were upregulated. These molecules were primarily expressed in the kidney tubule epithelial cells (*p* < 0.05, Figure 5E–G).

### 3.4. C/EBPβ–TFAM-Mediated NLRP3 Inflammasome Activation May Contribute to As-Induced Kidney Injury

**Figure 6:**
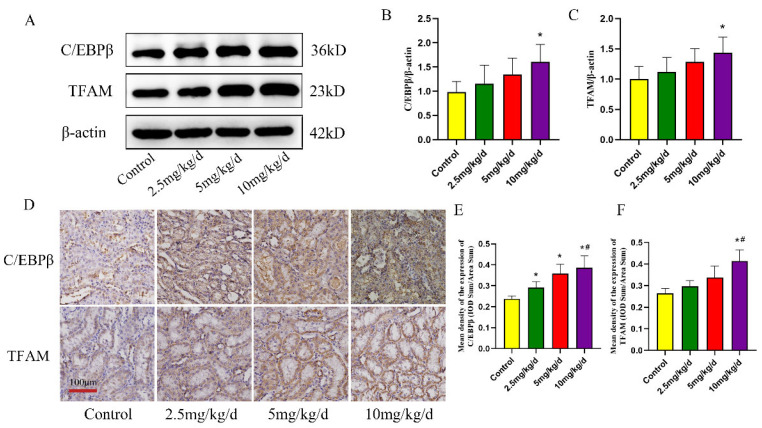
Expression of C/EBPβ and TFAM in the rat kidney. (**A**) Western blot detection of C/EBPβ and TFAM expression. (**B**,**C**) Quantitative analysis of C/EBPβ and TFAM protein expression, respectively. (**D**) Immunohistochemical detection of C/EBPβ and TFAM. (**E**,**F**) Quantitative analysis of C/EBPβ and TFAM immunohistochemical detection, respectively. * *p* < 0.05 compared with the control group, # *p* < 0.05 compared with the 2.5 mg/kg/d NaAsO_2_ group. Data are reported as means ±SD (*n* = 6).

To investigate the mechanism underlying As-induced activation of NLRP3 inflammasome, C/EBPβ and TFAM were examined. The expression levels of C/EBPβ and TFAM in the kidney tissues were detected by Wb and IHC. The expressions of C/EBPβ and TFAM in the kidney tissues of the rats in the As-exposed groups were upregulated (*p* < 0.05, Figure 6A–C). As expected, the C/EBPβ and TFAM expression results were consistent with the IHC analysis, and C/EBPβ and TFAM were mainly expressed in tubular epithelial cells in the kidneys (*p* < 0.05, Figure 6D–F).

## 4. Discussion

As is a common toxic metalloid element. Humans can ingest As through inhalation, food and drinking water. Long-term As exposure can cause multi-organ and multi-system damage. Damage to the kidneys, as the primary excretory organ of As, affects the excretion of As and its metabolites, leading to the accumulation of As in the body. This can result in systemic multi-organ damage [24]. In our previous studies, we demonstrated that As-exposed people have significantly higher levels of β2-MG, urinary NAG and urinary ALB. However, the mechanism was not clear. In recent years, the critical role of disturbed immune homeostasis and inflammatory responses in kidney-related diseases has garnered considerable interest among scientists [25,26]. Therefore, a rat model of sub-chronic As exposure was established in this study via the oral administration of NaAsO_2_. The aim was to assess the inflammation mechanisms underlying As-induced kidney impairment.

The kidney tissues of sub-chronic As-treated rats exhibited different degrees of injury, including glomerular vacuole-like changes, tubular dilatation and inflammatory cell infiltration [26]. The analysis of kidney function revealed that arsenic-treated rats had significantly higher daily excretion of β2-MG, NAG, and ALB via urine. β2-MG is a component of the histocompatibility complex and is present on the surface of nucleated cells. It is produced at a constant rate and freely filtered by the kidneys, completely reabsorbed by endocytosis on the proximal tubules, and then completely catabolized within the tubulocytes. Normally, only small amounts of β2-MG are present in the urine. However, when the kidney tubules become damaged or diseased, the urine β2-MG concentration increases. Therefore, elevated β2-MG in the urine signifies reduced tubular reabsorption and possible injury in kidney tubules [27]. In addition, under normal physiological conditions, trace levels of ALB pass through the glomerular basement membrane. Thus, elevated urinary ALB is a reliable diagnostic indicator for the early detection of nephropathy [28]. Another tubular injury marker is NAG which is expressed in a variety of tissues throughout the body and is not filtered through the glomerulus when healthy due to its relatively high molecular weight, and it was found to be a lysosomal enzyme and its elevation suggests kidney injury. NGAL is a secreted human lipid transport protein that is isolated from human neutrophils. Under normal physiological conditions, NGAL expression in the kidneys is extremely low. However, when the epithelial cells are damaged, the expression of NGAL increases significantly, especially in kidney injury [29]. It has been demonstrated that NGAL is a good predictor of kidney injury, reflecting the severity of kidney disease, and that elevated NGAL levels may be associated with damage occurring in medullary collaterals and distal tubules [30]. The results in this study revealed that NGAL expression in kidney tissues was considerably elevated after As treatment. This provides further confirmation of As-induced kidney injury in rats at the tissue level. Taken together, the above results suggest the presence of damaged (or disrupted) kidney function in As-treated rats.

As a key inflammatory response process in As-induced systemic multi-organ injury, our group previously found As-induced lung injury and collagen fibril deposition in rats via the activation of the HMGB1/RAGE-mediated proinflammatory response [23]. Similarly, a cytokine-mediated pro-inflammatory–anti-inflammatory imbalance is crucial in As-induced liver injury [31,32]. More importantly, the inflammatory response and cytokines can mediate kidney injury through various mechanisms, such as inflammatory cell infiltration, hemodynamic changes and endothelial dysfunction [33]. Several animal studies have indicated that increased IL-18 secretion promotes the development of kidney diseases [32,34]. In this study, the IL-18 and IL-1β expression levels in the kidney tissues of rats exposed to As were upregulated. This indicates that inflammatory responses are vital in As-induced kidney injury.

An inflammatory response is a protective immune response of an organism in reaction to exogenous stimuli. However, excessive inflammation can cause damage to the organism. Inflammasomes are receptors in the innate immune system that regulate the activation of caspase-1 and stimulate the inflammatory response. The NLRP3 inflammasome responds to various stimuli. Overactivation of the NLRP3 inflammasome can lead to the onset and progression of a variety of diseases, including diabetes mellitus, atherosclerosis, rheumatoid arthritis, ischemic heart disease, liver disease, kidney disease and so on [32]. The level of NLRP3 inflammasome in CKD patients may be affected by uremic toxins (UT), and the plasma UT level in CKD patients is significantly increased, which promotes the progression of CKD by inducing autophagy, inflammatory response and oxidative stress [35]. Activation of the NLRP3 inflammasome induces the synthesis and release of the inflammatory parameters IL-1β and IL-18 [36,37]. New evidence suggests that NLRP3 levels are considerably increased in the kidneys of chronic kidney disease (CKD) patients [14]. In this study, the expression of NLRP3, caspase-1, ASC and downstream inflammatory parameters were elevated in the kidneys of rats treated with As. This finding suggests that As induces NLRP3 inflammasome activation, which triggers an extracellular inflammatory response in the kidneys [38].

This study has revealed a novel role of TFAM in As-induced kidney injury in rats and has provided insight into the underlying mechanisms. In this in vivo model of As-induced rat kidney injury, TFAM was significantly increased. Notably, TFAM was primarily expressed in kidney tubular epithelial cells, with the IHC results showing brownish yellow granules. It is hypothesized that this may be due to the release of TFAM from inflammatory necrotic cells, which is recognized by DAMPs, and that TFAM may then mediate the As-induced inflammatory response in kidney tubular epithelial cells. It was also previously reported that knockdown of TFAM significantly attenuates the LPS-induced inflammatory response, suggesting that TFAM plays an important role in kidney epithelial cell inflammation and heat shock [39].

It has been shown that the interaction between C/EBPβ and TFAM activates NLRP3/caspase-1 and promotes the development of acute kidney injury [39]. However, the effect of C/EBPβ on TFAM in As-induced kidney injury in rats is yet to be understood. In this study, C/EBPβ was significantly upregulated in the As-treated rat model, with significant increases as a function of increases in the dose of As treatment. It is speculated that C/EBPβ may target the promoter region of TFAM and promote its expression. The above results suggest that sub-chronic As exposure triggers inflammatory responses in rat kidney tissues. It is speculated that the underlying mechanism may involve the promotion of TFAM expression by As via the promotion of transcription factor C/EBPβ expression, followed by the binding of C/EBPβ to the promoter region of TFAM; TFAM is then recognized by the NLRP3 inflammasome as an injury-related molecular pattern, mediating the activation and release of downstream inflammatory parameters. This ultimately leads to kidney tissue injury. This hypothesis is supported by a previous study that reported that injection of TFAM into the brains of male Sprague-Dawley rats upregulated a range of pro-inflammatory factors [18]. Similarly, extracellular TFAM increased the expression of pro-inflammatory factors and NLRP3 in microglia in a concentration-dependent manner [39].

A shortcoming of this study is that the expression of related molecules following As exposure was only tentatively determined. Moreover, the regulatory relationships between molecular networks are also speculative based on existing studies. The specific regulatory relationships are yet to be verified in knockout mice or in vitro experiments. In addition, the relatively small sample size may limit the biological significance of some of the results of this study. Future studies with larger sample sizes are warranted. This would also allow for the comparison of differences in kidney injury between female and male rats. Finally, the present study only examined the expression of representative inflammatory factors, i.e., IL-1β and IL-18. A comprehensive assessment of biologically inactive pro-IL-1β and pro-IL-18 is necessary in the future.

## 5. Conclusions

**Figure 7:**
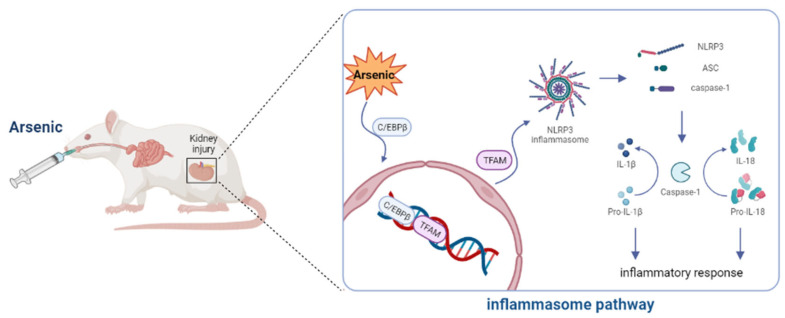
The possible mechanism underlying As-induced kidney injury in rats.

In brief, this study discovered that As exposure triggers inflammatory infiltration in rat kidney tissue. The mechanism might be related to C/EBPβ–TFAM-mediated activation of NLRP3 inflammatory vesicles. Targeting C/EBPβ and TFAM to modulate inflammatory responses is a potentially viable intervention strategy to address As-induced kidney injury (Figure 7).

## Data Availability

Data will be made available on request.
